# A nomogram for predicting sclerotherapy response for treatment of lymphatic malformations in children

**DOI:** 10.1186/s40001-022-00844-3

**Published:** 2022-10-21

**Authors:** Zhiping Wu, Yun Zou, Ronghua Fu, Pingliang Jin, Hua Yuan

**Affiliations:** Department of Plastic Surgery, Jiangxi Provincial Children’s Hospital, Nanchang, China

**Keywords:** Lymphatic malformation, Sclerotherapy, Pingyangmycin, Polidocanol, Nomogram

## Abstract

**Purpose:**

In this manuscript, we purposed to identify the prognostic factors for treatment of lymphatic malformations in children using polidocanol foam combined with pingyangmycin and to construct nomogram for predicting sclerotherapy response.

**Methods:**

A retrospective analysis of 77 children having LMs who underwent sclerotherapy using polidocanol foam combined with pingyangmycin under ultrasound display from January 2017 to April 2020 was done. The clinical response was graded as excellent (≥ 90%), good (≥ 50%, < 90%), and poor (< 50%). More than 50% was considered as acceptable response. Prognostic factors were identified by Pearson’s Chi-square or Fisher’s exact test and multivariable logistic regression model was used to construct a nomogram to predict sclerotherapy response. The discrimination and calibration of nomogram were verified through the receiver operating characteristic cure and calibration plots.

**Results:**

The mean number of treatment sessions was 3.1 (range, 1–6). Among 77 patients, 58 patients (75.3%) had excellent response to treatment (≥ 90%) and 68 patients (88.3%) had an acceptable response (≥ 50%, < 90%). Clinical disfigurement (*P* = 0.014), skin discoloration (*P* = 0.040), morphological subtype (*P* < 0.001) and extent of the lesion (*P* < 0.001) correlated with clinical response to sclerotherapy in LMs. Sclerotherapy response was predicted through nomogram constructed in this study, which shows good calibration and discrimination. Also, focal lesion and macrocystic or mixed morphological subtype lesion were seen more often in lower number of treatment sessions among the patients with excellent response.

**Conclusions:**

An acceptable response to sclerotherapy using polidocanol foam combined with pingyangmycin was achieved in majority of LMs in children with extremely low complication rates. Nomogram based on the prognostic factors of sclerotherapy response for LMs in children was shown to possess an excellent performance to predict the probability of LMs sclerotherapy response.

## What is known:

• A plenty of sclerosants had been proposed to sclerotherapy of lymphatic malformations in children.
• Microcystic lesions have worse outcomes compared with macrocystic or mixed lesions.

## What is new:

• Polidocanol foam combined with pingyangmycin appears to be an effective and safe treatment for lymphatic malformations in children.
• Clinical disfigurement, skin discoloration, morphological subtype and extent of the lesion correlated with clinical response to sclerotherapy in lymphatic malformations in children.

## Introduction

The International Society for the Study of Vascular Anomaly (ISSVA) classification is widely recognized and divides vascular abnormalities into vascular tumors and vascular malformations [1, 2]. The size of the malformation may increase, in contrast to vascular tumors which are characterized by spontaneous regression. Vascular malformations contain two subtype disorders: high-flow disorders (arteriovenous malformations and arteriovenous fistula) and low-flow lesions (venous and lymphatic malformations). Lymphatic malformations (LMs) results from errors in the embryonic development of the lymphatic system and is estimated to occur in about 1 in 2000 live births, diagnosed mainly at birth or within 2 years of birth. LMs can occur in any part of the body, but 75% of LMs affect the head and neck region [3].

According to ISSVA classification, LMs are subdivided into macrocystic lesions (cysts > 2 cm), microcystic lesions (cysts < 2 cm) and mixed lesions [4]. Symptoms vary depending on the size, location and anatomy of the LMs involved. Small lesions may be asymptomatic until infection or bleeding develops, while complex lesions may be accompanied by pain, edema, deformation and even airway compression. Patients can suffer from mild swelling to life-threatening airway obstruction, macroglossia, impaired oral feeding, vision loss, overgrowth of the mandible, esthetic defects and pain, when lesion locate in the head and neck [5, 6].

The main aim of LMs management is to correct functional impairment and esthetic deformation. Prior to the development of minimally invasive methods, the primary means of treatment for LMs was surgery; whereas, it was correlated with a number of complications such as seroma, infection, hematoma/hemorrhage, nerve damage and scar deformity [7, 8]. Currently, there are a variety of treatment options include watchful waiting, medication (primarily sirolimus or sildenafil), sclerotherapy, laser/radiofrequency ablation, or a combination of the above [9–11]. Sclerotherapy is becoming the first-line treatment of choice for LMs due to relatively few complications and low recurrence rates.

Although LMs has widely treatment, a standardized grading scale to assess outcomes is still absence and there are a few research in this area. Serres et al. proposed a preoperative staging scale for LMs in 1995 to predict prognosis and outcome, but this scale only focused on surgical intervention based on lesion site [12]. And there are studies developing a consensus statement or radiologic grading system for evaluating the treatment of LMs [13, 14]. However, these studies mainly report the lesion on head and neck, and include both adults and children. Recently, one study investigated clinico-radiologic predictors to predict the clinical outcome, which evaluated on the sclerotherapy response in low-flow vascular malformations [15]. At present, driven by clinical needs, there is an urgent need to establish models to predict prognostic probability and guide targeted treatment strategy. Therefore, it is necessary to establish a quantitative prediction model to predict sclerotherapy response in LMs patients, so as to help clinicians make specific treatment recommendations for patients.

This study purposed to identify prognostic factors for sclerotherapy response of LMs in children. In addition, construction of a reliable and reproducible nomogram for clinical treatment outcomes of LMs, which offers the ability to both refine reporting standards and clarify communication between treating physicians.

## Methods

### Patient enrollment

From January 2017 to December 2019, 77 children diagnosed with LMs were recruited in the Department of Plastic Surgery, Jiangxi Provincial Children's Hospital.

Inclusion criteria comprised: patients with LMs. According to ISSVA definition, physical examination, Doppler ultrasonography, and magnetic resonance imaging confirmed the diagnosis. Exclusion criteria included: LMs patients with a history of treatment; patients with severe systemic disease who cannot tolerate general anesthesia; patients are allergic to polidocanol or pingyangmycin.

This research was granted by the Institutional Review Committee of ethics Committee of Jiangxi Provincial Children’s Hospital, and all guardians signed informed consents. This study was conducted according to the ethical guidelines of the Declaration of Helsinki.

### Sclerotherapy regimen

All patients experienced a complete clinical examination, Doppler ultrasonography or magnetic resonance imaging. Preparation of 3% (60 mg) polidocanol (Aethoxysklerol; Kreussler Pharma, Wiesbaden, Germany), 8 mg pingyangmycin (Jilin Aodong Pharmaceutical Group Co., Ltd., Jilin, China). The sclerosing foam are directly injected in the field using a 1:3 liquid to gas ratio. Two syringes are connected by a three-way stopcock, one filled with polidocanol and one filled with air; the foam is made by mixing two syringes having multiple channels as stated by Tessari.

All operations were undergone in the operating room in general anesthesia condition, which could offer adequate compliance in pediatric patients. After proper sterilization, a single transfusion needle is inserted percutaneous using a double or multiple syringe system under ultrasound display. When the needle located in the cyst, the polidocanol (POL) foam and pingyangmycin was injected, respectively, after aspiration of an adequate amount of the lymphatic fluid. Applied local pressure to the injection site and observed carefully for 15 min. A compression pads should be used for the first 3 days. All patients were informed to follow-up or treatment followed 1 month later. Generally, one to three sessions comprised one treatment cycle, depending on the individual's response, the size of lesion. Until the lesion was dissolved and no lymphatic fluid was drained, the treatment cycle was considered finished. The maximum dose of pingyangmycin and polidocanol should not exceed 0.5 mg/kg and 2 mg/kg, respectively. After completion of treatment, patients were advised to review every 12 weeks.

### Outcomes

Outcomes included response to treatment, number of treatment sessions, and complications of post-treatment. The post-treatment complications and number of treatment sessions were documented. The treatment response, which was based on clinical examination and imaging findings after each treatment, was classified into excellent response (completely cured, lesion reduction ≥ 90%), good response (significantly improved appearance, lesion reduction ≥ 50%, < 90%), and poor response (improved appearance, lesion reduction < 50%). And excellent and good response were defined as an acceptable response. All patients were followed up for an average of 16.5 months.

### Statistical analysis

Quantitative data were displayed by means and standard deviation or median and range. And categorical data were described by number and percentage (N, %). Continuous data were analyzed by independent t test and categorical data were compared using Pearson’s Chi-square or Fisher’s exact test where appropriate. The statistically significant factors for therapeutic response were then used to constructed a multivariable logistic regression model. Based on the multivariable logistic regression model, a predictive nomogram for LMs response was developed. Receiver operating characteristic (ROC) and calibration curves were used to assess the efficacy. Statistically significant levels were two-tailed and set at P < 0.05. Statistical analysis was performed using IBM Statistical Package for the Social Sciences (SPSS) version 22.0 Windows software package. The nomogram and ROC curve were drawn using the “rms” package in R version 3.4.1.

## Results

### Clinical features

A total of 77 patients (38 males and 39 females) were included; mean age: 3.3 ± 1.2 years (1 day to 18 years); direct intra-focal sclerotherapy was performed with polidocanol and pingyangmycin. The most commonly affected site was head and neck region (*n* = 35, 45%), followed by extremities (*n* = 17, 22%), thorax (*n* = 12,16%), and abdomen and pelvis location (*n* = 10, 13%). The spectrum of clinical symptoms included swelling (71% of patients), pain (39% of patients), disfigurement (55%), skin discoloration (53%), restricted range of motion (13%), and bleeding (42%). 15 patients (19%) were diffuse and involved two or more anatomic locations. In the morphological subtypes, macrocystic LMs (40%) were the most common patterns.

In this study, a total of 214 treatment sessions with sclerotherapy were performed in the 77 patients. The mean number of treatment sessions was 3.1, ranging from 1–6 sclerotherapy sessions per patient. 55% and 18% patients received one and two procedures during the treatment sessions, respectively. The average follow-up period was 18 (range, 6–30) months (Table 1).

**Table 1:** Demographic and clinical characteristics of patients

	Number	Percentage (100%)
Age		
< 1 year	40	52
1–2 years	14	18
> 2 years	23	30
Gender		
Male	38	49
Female	39	51
Swelling		
Yes	55	71
No	22	29
Pain		
Yes	30	39
No	47	61
Disfigurement		
Yes	42	55
No	35	45
Discoloration		
Yes	41	53
No	36	47
Bleeding		
Yes	32	42
No	45	58
Limited motion		
Yes	10	13
No	67	87
Morphological subtypes		
Macrocystic LMs	31	40
Microcystic LMs	21	27
Mixed LMs (macrocystic and microcystic)	25	33
Anatomical location		
Head and neck	35	45
Thorax	12	16
Abdomen and pelvis	10	13
Upper extremity	8	10
Lower extremity	9	12
Abdomen/pelvis/lower extremity	3	4
Extent		
Focal	62	81
Diffuse	15	19

	Mean	Range
Age (years)	3.3	1 day—18 years
Treatment sessions (No.)	3.1	1–6
Follow-up period (months)	18	6–30

### Clinical response

In patient-level evaluations, 58 patients responded excellent to treatment (group A), 10 responded good to treatment (group B), and 9 responded poor to treatment (group C) at the end of the study period. Thus, 75.3% of patients had an excellent response and 88.3% patients had an acceptable response. Disfigurement (*P* = 0.014), skin discoloration (*P* = 0.040), morphological subtype (*P* < 0.001) and extent of the lesion (*P* < 0.001) had significant influence on the clinical efficacy of each patient (Table 2). The clinical response was better in macrocystic and mixed lesions compared with microcystic lesions ((*P* < 0.001 and *P* = 0.046, respectively). And patients with diffuse lesion had a worse clinical response compared with local lesion. Absence of skin discoloration, disfigurement correlated with an excellent response.

**Table 2:** Excellent responders (group A vs groups B and C) with respect to demographic and clinical characteristics

	Groups B and C (19)	Group A (excellent responders) (58)	*P* value
Age			0.624
< 1 year	11	29	
1–2 years	4	10	
> 2 years	4	19	
Gender			0.391
Male	11	27	
Female	8	31	
Swelling			0.738
Yes	13	42	
No	6	16	
Pain			0.827
Yes	7	23	
No	12	35	
Disfigurement			0.014
Yes	15	27	
No	4	31	
Discoloration			0.04
Yes	14	27	
No	5	31	
Bleeding			0.956
Yes	8	24	
No	11	34	
Limited motion			0.713
Yes	2	8	
No	17	50	
Morphological subtypes			< 0.001
Macrocystic LMs	0	31	
Microcystic LMs	12	9	
Mixed LMs (macrocystic and microcystic)	7	18	
Extent			< 0.001
Focal	9	53	
Diffuse	10	5	

Among the patient with excellent response, there was also a significant higher number of treatment sessions per patient in patients with microcystic lesion (median 4, range 3–6) over the study period compared with patients with macrocystic (median 1, range 1–6; *P* = 0.034) or mixed lesion (median 3, range 2–6; *P* = 0.042). Also, the patient with local lesion (median 1, range 1–3) had a significantly lower number of sessions compared with diffusion lesion (median 3, range 1–6; *P* = 0.026). But there was no significantly difference in skin disfigurement and discoloration in the number of sessions performed (Table 3).

**Table 3:** Sclerotherapy sessions (patient of excellent response) with respect to clinical characteristics

	Median treatment sessions(range)	*P*
Disfigurement		0.064
Yes	2 (1–6)	
No	2 (1–5)	
Discoloration		0.078
Yes	3 (1–5)	
No	2 (1–5)	
Extent		0.026
Local	1 (1–3)	
Diffuse	3 (1–6)	
Morphological subtypes		
Macrocystic LMs	1 (1–3)	0.034
Mixed LMs (macrocystic and microcystic)	3 (2–6)	0.042
Microcystic LMs	4 (3–6)	reference

### Nomogram construction

**Fig. 1:**
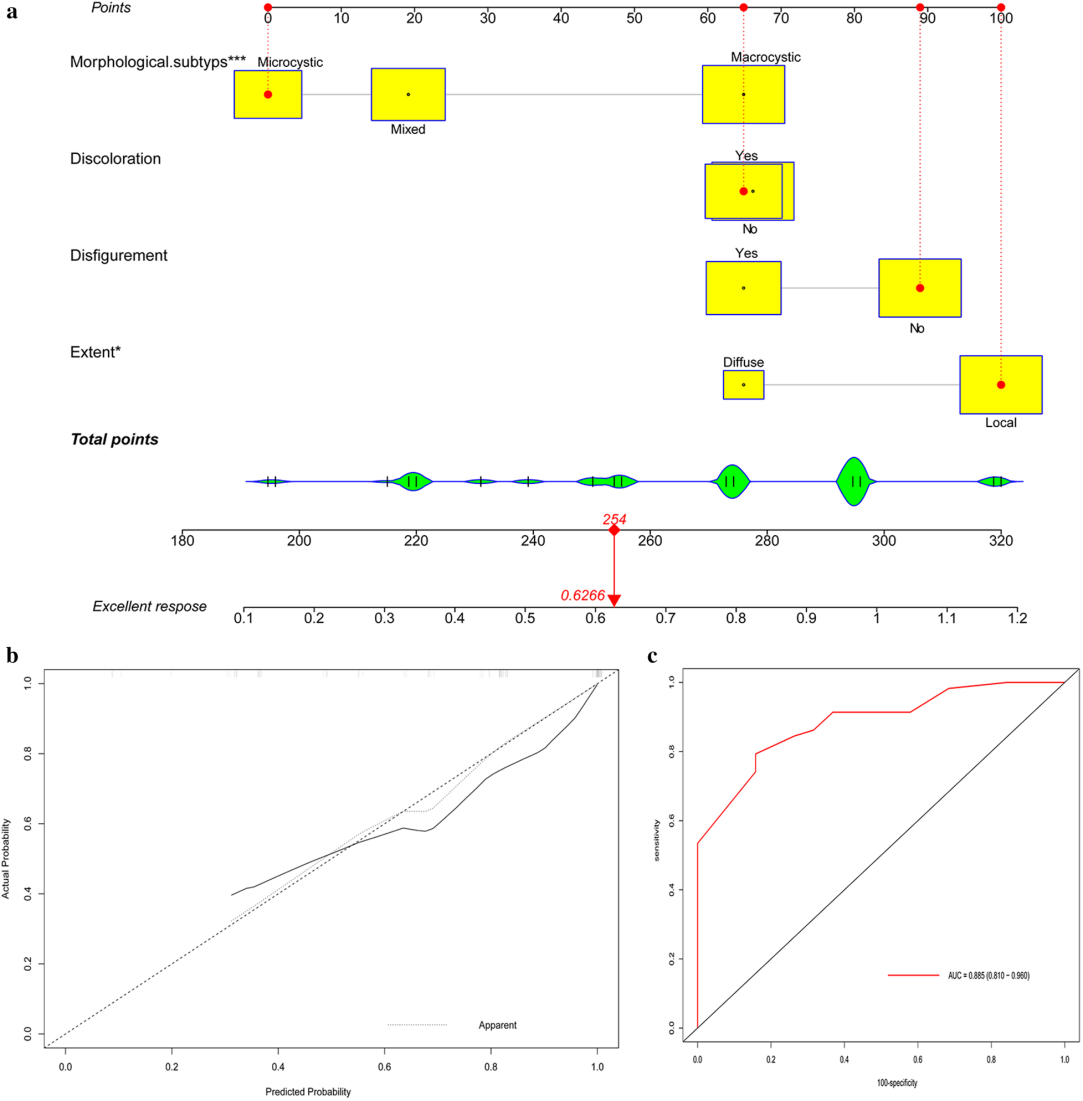
A nomogram for predicting excellent response in two treatment circles (**A**), calibration curve (**B**), and ROC curve (**C**) for assessing the calibration and discrimination of the nomogram in predicting excellent response

All significant factors for clinical response were integrated into the nomogram to predict the sclerotherapy response probability (Fig. 1a). As shown in Fig. 1b, the calibration curve shows that the predicted probability is in good agreement with the observed probability. In addition, the ROC curve showed good discrimination, with nomogram predicting a response AUC of 88.5% (95% CI 81.0–96.0%) (Fig. 1c).

### Procedure-related complications

Regarding the safety of sclerotherapy with polidocanol and pingyangmycin, no serious complications were observed in this study. Fifty-four patients (77%) developed mild swelling after sclerotherapy, with a mean duration of swelling of 5.4 days (range 3–10). One child developed significant swelling after the injection, but the symptoms resolved within 2 weeks, with no signs of infection or other identifiable complications. Systemic complications: one child developed mild fever below 38.5 ℃ 3 days after sclerotherapy. In addition, one child developed gastrointestinal reactions and sleep disturbances, respectively. These minor complications resolved within 1 week without special treatment.

## Discussion

To our knowledge, there are sparse studies that have explored prognostic factors of sclerotherapy in LMs and a predictive nomogram prediction model is absence at present; meanwhile, most researches have focused on the efficacy of each sclerosing agents [16–18]. In addition, many studies only compared the efficacy of various sclerosants in children with LMs [19, 20]. However, researches mainly focused on the prognostic variables for LMs with sclerotherapy were relative sparse. In this study, we assessed therapeutic effect and investigated the prognostic factors for sclerotherapy response of LMs in children with a large number of samples. The nomogram constructed in our study will help predict the long-term response of LMs in children, and help clinicians make individualized treatment strategy and communicate the prognosis with their families.

Numerous sclerosing agents have been proposed over the years. In recent years, microfoam injections have become increasingly popular because of their unique properties and ease of management. Particularly, POL is a non-ionic surfactant sclerosing agent that directly damages vascular endothelial cells by activating cellular calcium signaling and nitric oxide pathways, thereby reducing malformation volume. POL foam replaces blood rather than diluting it, and tightly controls the concentration of agents in the blood vessels and distributes them evenly throughout the lumen.

POL foam sclerotherapy for vascular malformations has been well documented in previous studies.

One study demonstrated that POL foam could diminish the size of lesion in 24 vascular malformations patients [21]. Yamaki et al. found that sclerotherapy with POL foam was an effective and safe treatment in 32 patients with LMs [22]. The study showed that 88% of patients experienced excellent and moderate responses, while intralesional bleeding was observed in 13% of patients. In general, polidocanol is considered as a safe but relatively weaker potent sclerosant compared to other sclerosants. Moreover, one single sclerosing agent could not obtain excellent results in many patients.

Pingyangmycin is extracted from bleomycin (bleomycin A5) and is becoming one popular sclerosing agents [23]. Pingyangmycin and bleomycin contains similar chemical structure and composition and are common drugs to vascular malformations and a kind of tumors [24]. Pingyangmycin and bleomycin have similar curative effect, but it has less complication and low cost [25]. Plenty of studies found that pingyangmycin has an excellent therapeutic effect on deep microcystic LMs in the face and tongue [24, 26]. In the case of macrocystic LMs, one study showed that 84.38% of patients had a satisfactory final recovery in 32 participants who treated with injections of pingyangmycin [27]. Furthermore, Luo and Gan demonstrated that pingyangmycin was an effective and safe sclerosing agent in LMs [28]. According to the above studies, pingyangmycin was an ideal sclerosant to combine with POL foam to enhance therapeutic effect. In addition, pingyangmycin and polidocanol are a safer choice, because these two substances are stable in physical and chemical characteristics. Meanwhile, it has been reported that chemical reaction could not happen when them are mixed [29]. Thus, this study evaluated the therapeutic effect of polidocanol foam combined with pingyangmycin in LMs and developed a predictive nomogram for LMs response.

This large, retrospective study showed the effectiveness of sclerotherapy using polidocanol foam and pingyangmycin sclerosants in managing patients with LMs in children. Meanwhile, we found the skin discoloration, disfigurement, morphological subtype and extent of the lesion had significant influence on the clinical efficacy of each patient. We also found, among the patient with excellent response, morphological subtype and extent of the lesion had significant influence on the number of sessions performed per patient during the study period.

Identification of prognostic factors is of great significance to guide individualized treatment and improve treatment response. There are studies found that well-defined margin, female gender, macrocystic type and phlebographic characteristic were variables correlated with the therapeutic effect in patients with low-flow vascular malformations or LMs [15, 30, 31]. At the present study, we confirmed four prognostic factors for LMs patients with sclerotherapy using polidocanol foam and pingyangmycin, and the result was partly similar to the previous studies. LMs patients achieved a significantly better sclerotherapy response if they had the following characteristics: absence of skin discoloration, absence of disfigurement, macrocystic, and focal lesion. At present, few studies have focused on the prognostic variables for LMs with sclerotherapy. As reported in this study, disfigurement was a factor associated with LMs efficacy. The patients without of disfigurement were significantly better than that of patients with it. In addition, skin discoloration was identified that correlated with sclerotherapy response in LMs, and the efficacy of patients without skin discoloration was much better than that of other patients. But, the influence of these two factors on efficacy of LMs needs to be further identified in the further studies. Some studies indicated that the morphological subtypes of LMs affected sclerotherapy response [20, 32, 33]. We found that the clinical response was better in macrocystic and mixed lesions compared with microcystic lesions. Various reasons may lead to this result. One reason may be the small size of the cyst or channel and poor diffusion of the sclerosants within the lesion. Perhaps more critically, there is a relatively large soft tissue component compared to small cysts in the microcytic lesions. Therefore, even when cysts dissolve or shrink, there are still residual soft tissue masses that cannot be treated with sclerotherapy [34]. What’s more, our study found that focal lesion were independent predictors of good response to sclerotherapy. As we known, venous vascular malformations (VMs) and LMs belong to the low-flow vascular malformations, so they have the same therapeutic characteristics in some area. In VMs, Goyal et al. divided VMs into three categories according to MRI results of VMs size and marginal morphology [35]. They reported that patients with VMs lesions greater than 5 cm or with ill-defined boundaries responded poorly to percutaneous sclerotherapy.

The limitations of this study include retrospective design not covering all LMs patients treated with sclerotherapy using polidocanol foam and pingyangmycin sclerosants and a lack of standardized assessment tool to compare our results with other reports.

## Conclusions

In summary, this study demonstrates that sclerotherapy using polidocanol foam combined with pingyangmycin appears to be an effective and safe treatment for LMs in children and some prognosis factors were confirmed. Nomogram basing on these prognostic factors was established to predict clinical response in children with LMs, which has good calibration and discrimination ability. The nomogram could help clinicians to make individualized predictions and provide targeted treatment recommendations for patients. In addition, inferring from our observation that macrocystic morphological subtype and focal of the lesion had significantly lesser number of sessions performed per patient.

## Abbreviations

ISSVA: The International Society for the Study of Vascular Anomaly
LMs: Lymphatic malformations
POL: Polidocanol
ROC: Receiver operating characteristic
VMs: Venous vascular malformations
